# Estimating absolute indoor density of *Aedes aegypti* using removal sampling

**DOI:** 10.1186/s13071-019-3503-y

**Published:** 2019-05-21

**Authors:** Edgar Koyoc-Cardeña, Anuar Medina-Barreiro, Azael Cohuo-Rodríguez, Norma Pavía-Ruz, Audrey Lenhart, Guadalupe Ayora-Talavera, Mike Dunbar, Pablo Manrique-Saide, Gonzalo Vazquez-Prokopec

**Affiliations:** 1 Unidad Colaborativa de Bioensayos Entomológicos, Campus de Ciencias. Biológicas y Agropecuarias, Universidad Autónoma de Yucatán, Mérida, Yucatán Mexico; 2 Centro de Investigaciones Regionales “Dr. Hideyo Noguchi”, Universidad Autónoma de Yucatán, Mérida, Yucatán Mexico; 3 Entomology Branch, Division of Parasitic Diseases and Malaria, Center for Global Health, Centers for Disease Control and Prevention, Atlanta, GA USA; 4 Department of Environmental Sciences, Emory University, Atlanta, GA USA

**Keywords:** Sampling, Entomology, Population abundance

## Abstract

**Background:**

Quantification of adult *Aedes aegypti* abundance indoors has relied on estimates of relative density (e.g. number of adults per unit of sampling or time), most commonly using traps or timed collections using aspirators. The lack of estimates of the sensitivity of collections and lack of a numerical association between relative and the absolute density of adult *Ae. aegypti* represent a significant gap in vector surveillance. Here, we describe the use of sequential removal sampling to estimate absolute numbers of indoor resting *Ae. aegypti* and to calculate calibration coefficients for timed Prokopack aspirator collections in the city of Merida, Yucatan State, Mexico. The study was performed in 200 houses that were selected based on recent occurrence of *Aedes*-borne viral illness in residents. Removal sampling occurred in 10-minute sampling rounds performed sequentially until no *Ae. aegypti* adult was collected for 3 hours or over 2 consecutive 10-minute periods.

**Results:**

A total of 3439 *Ae. aegypti* were collected. The sensitivity of detection of positive houses in the first sampling round was 82.5% for any adult *Ae. aegypti*, 78.5% for females, 75.5% for males and 73.3% for blood-fed females. The total number of *Ae. aegypti* per house was on average ~5 times higher than numbers collected for the first sampling round. There was a positive linear relationship between the relative density of *Ae. aegypti* collected during the first 10-min round and the absolute density for all adult metrics. Coefficients from the linear regression were used to calibrate numbers from 10-min collections into estimates of absolute indoor *Ae. aegypti* density for all adults, females and males.

**Conclusions:**

Exhaustive removal sampling represents a promising method for quantification of absolute indoor *Ae. aegypti* density, leading to improved entomological estimates of mosquito distribution, a key measure in the assessments of the risk pathogen transmission, disease modeling and the evaluation of vector control interventions.

## Background

If all individuals in a population cannot be counted, they must be sampled [1]. This basic principle represents the cornerstone of ecological field studies and provides the basis for estimating the relative or absolute numbers of individuals within a population or community [1, 2]. Many factors, including ecological, economic and statistical, influence the methodologies employed to estimate population size, which broadly include mark-recapture, catch-per-unit-effort (CPUE, e.g. trapping, timed collections), removal sampling, distance sampling and quadrat methods [1, 2]. When populations have to be monitored regularly or at multiple locations, simpler (and often less expensive) and potentially less accurate methods such as CPUE are generally preferred to the more costly mark-recapture approaches [1]. The use of CPUE is common practice in medical entomology [3]. Some examples include the use of passive or active traps (e.g. CDC light traps, ovitraps, adult resting boxes), timed collections (e.g. adult mosquito aspiration, kissing bug timed manual collections) and immature habitat sampling (e.g. *Aedes* pupal surveys, tick dragging). Irrespective of the method, all such approaches provide a measure of relative abundance (also called density), in which the number of collected individuals is a function of the time or effort employed to collect them. Such estimates are prone to bias for multiple reasons, including differences between the collectors themselves, heterogeneity of captures across space, and sensitivity of the sampling methods in situations of low population abundance [1, 2]. More often than not, such biases are not appropriately quantified, which limits the validity of estimates of relative density, particularly in situations in which vector abundance is low or spatially heterogeneous. In order for a sampling methodology to be robust and valid, measures of the association between the relative sample and the absolute density are desired [1, 2, 4, 5].

*Aedes aegypti* rest primarily indoors [6], where they frequently and preferentially bite humans [7]. Compared to other vector mosquitoes (e.g. *Culex quinquefasciatus*), *Ae. aegypti* is considered a low abundance species [8]. Multiple approaches have been implemented to estimate *Ae. aegypti* density and population size, with mark-recapture experiments [9–11], the innovative use of the ratio of wild-type to *Wolbachia*-infected released mosquitoes [12, 13] or statistical/mathematical models fitted to field data [14, 15] as the most commonly used. Mark-recapture (whether with dust or *Wolbachia*) studies point to an average of 5–10 females per premise as the density of *Ae. aegypti* during peak-transmission periods [11–13]. Extensive indoor aspiration sampling performed in Iquitos, Peru, collected <10 adults per house, on average, during periods of high virus transmission [16]. In Cairns, Australia, Williams et al. [14] used pupal productivity data fitted to a mathematical model of *Ae. aegypti* productivity to estimate an average of 4–23 female *Ae. aegypti* per premise during the period of arbovirus transmission (wet season). All such approaches relied on sampling methods such as BG sentinel traps, which may also be prone to error. For instance, using a mini mark-release study design, Johnson et al. [17] estimated that the BG sentinel trap captures ~20–30% of adults outdoors, providing a measure of the sensitivity or sampling rate for the traps. This is, to our knowledge, the only published attempt of calibration of relative to absolute abundance for any adult *Ae. aegypti* sampling method.

Timed adult aspiration (the use of motorized vacuums by an operator to capture resting and flying mosquitoes indoors during a defined period) is considered a gold standard for indoor adult *Ae. aegypti* sampling [8, 18]. Used primarily in research, adult aspiration provides a relatively unbiased measure of mosquito relative abundance (i.e. collecting both males and females as well as fed and unfed females) when conducted for ~10-minutes per house [19–22]. This rapid measure of relative density may be prone to collector variability, or to differential capture rate as a function of vector density or the size and complexity of premises. Given no study has yet quantified the sensitivity of sampled and absolute estimates of *Ae. aegypti* density using aspiration devices, the magnitude of such potential sources of bias is unknown. To address this gap, we performed a field study to calibrate Prokopack [22] aspirator collections using correction coefficients derived from comparing sampled to absolute density estimated from sequential removal sampling. Removal sampling, where the sequential removal of individuals from the population using constant effort leads to a reduction in the catch per unit effort, can be used to estimate total population size [1]. The method assumes that: (i) the population is closed; (ii) the probability of each individual being caught is constant; and (iii) all individuals have the same probability of being collected on any given sample [1]. The original method involved fitting simple maximum likelihood regression models to data on the number caught on the *t* occasion *versus* the total catch up to occasion *t* − 1, allowing estimating initial population size when *t* = 0 [1]. However, if the population is depleted through the sampling procedure, total catch (rather than linear regression estimates) can be used to estimate absolute abundance. The removal method has been useful for estimating population size for fisheries (e.g. [23–25]) and was applied to estimate the population size of *Ae. aegypti* in Kenya [9], *Anopheles* spp. in Pakistan [26] and the efficiency of *Ixodes scapularis* population size and dragging efficiency [27], but not yet to associate indices of relative and absolute mosquito density.

## Methods

The study was performed in the city of Merida (population ~1 million), Yucatan State, Mexico. Merida is highly endemic for dengue [28] and other *Aedes*-borne viruses [29], which in the city are transmitted solely by *Ae. aegypti* [29, 30]. A total of 200 houses located in an *Aedes*-borne disease transmission hot-spot area of Merida [29] were included in this study. Only houses with a recent (within 1 month) occurrence of a symptomatic case of dengue, chikungunya or Zika (based on information from the local Ministry of Health, MOH) were included. The study ran for two transmission seasons to achieve the desired target number of 200 houses. Indoor mosquito collections were performed during the period of most intense virus transmission (June–December). After obtaining informed consent from householders, exhaustive adult mosquito collections using Prokopack aspirators [22] were conducted using removal sampling. Mosquitoes were sequentially collected from each house using constant effort and at pre-defined intervals. Each house was visited by a team of three entomologists trained in the collection of adult mosquitoes using the Prokopack aspirator. The removal sampling collection sequence was as follows: one of the entomologists entered the house and collected mosquitoes resting in all rooms, including the kitchen and bathroom, for a period of 10 min. A timer was used to make sure collections ended at the 10-min mark, at which point the collector exited the house and gave the aspirator collection cup to a supervisor, who immediately sent a second collector inside the house to perform another 10-min collection round. This collection sequence continued for 3 h or until no *Ae. aegypti* were collected for two consecutive rounds, whichever occurred first. The supervisor was the only team member who knew whether *Ae. aegypti* was collected during each sampling round. This procedure blinded collectors and provided less opportunity for bias. We chose 10 min as the sampling time because it is the average sampling duration of a standard urban home in Merida [30] and elsewhere [16]. As collection proceeded, each aspirator collection cup was labeled with the house code and the collection sequence number. The collectors alternated who performed the first 10-min sampling at each house. After all entomological collections were complete, a survey was administered to the household head asking for basic information about the home (size, number of residents, presence of screens, etc). All collected mosquitoes were transferred to the laboratory, where they were knocked down in a −20 °C freezer for 10 min, and then sexed and identified to species following standard keys. Female *Ae. aegypti* were classified by their engorgement status following a categorical score [31] and then dissected to separate their head from the rest of the body for future virus testing.

### Data analysis

We focused analyses on measures of total *Ae. aegypti* adults, *Ae. aegypti* females, blood-fed *Ae. aegypti* females and *Ae. aegypti* males. A binomial generalized additive mixed model (GAMM) was fitted to the presence of *Ae. aegypti* in each house for each 10-min collection round. The model included infestation on each sampling round (presence = 1, absence = 0), with sampling round as a non-linear term (continuous variable, set in 10-min increments) and house ID as random intercept.

Absolute density per house was calculated in two ways: as the sum of *Ae. aegypti* collected across all sampling rounds (named total catch) or following the removal sampling equation method developed by Carle and Strub [16]. The regression method, also called maximum weighted likelihood method, fits a regression line to the number of mosquitoes caught on the *ith* sample, as a function of the total catch up to *ith* − 1. The equations of the method are: $M = \mathop \sum \nolimits_{i = 1}^{k} \left( {k - i} \right)_{{u_{i} }}$ where *k* is the number of samples taken and *u*_*i*_ the number of animals caught in the *ith* sample. The population size, *N*, is calculated as the smallest integer greater than the total catch, *T*, that satisfies the following inequality: $\left( {\frac{N + 1}{N - T + 1}} \right)\left( {\frac{kN - M - T + 0.5k}{kn - M + 1 + 0.5k}} \right)^{k} \le 1$. The standard error in this inequality is calculated using maximum likelihood [32]. We used the *removal* function from the R package FSA [33] to calculate *N* using the Carle and Strub method. We performed simple linear regression analysis to compare *N* (regression method) to the total catch, and assess the relevance of both approaches to estimate absolute density.

Mann-Whitney test was used to evaluate the difference in capture rate or sensitivity (percentage of *Ae. aegypti* collected in first 10-min collection round divided by the total catch across two levels of vector abundance; low was defined as ≤10 *Ae. aegypti*/house, high was defined as >10 *Ae. Aegypti*/house). Simple linear regressions calibrated the relative abundance to absolute abundance values. Maximum likelihood was used to fit the regression equation to the data. To assess the effect of any household characteristics influencing model fit, we performed a multiple linear regression including variables such as household size, presence of mosquito screens and recent use of insecticides. All analyses were performed within the R programing environment (https://www.r-project.org/) and GAMMs were run using the *lme4* package [34].

## Results

**Fig. 1:**
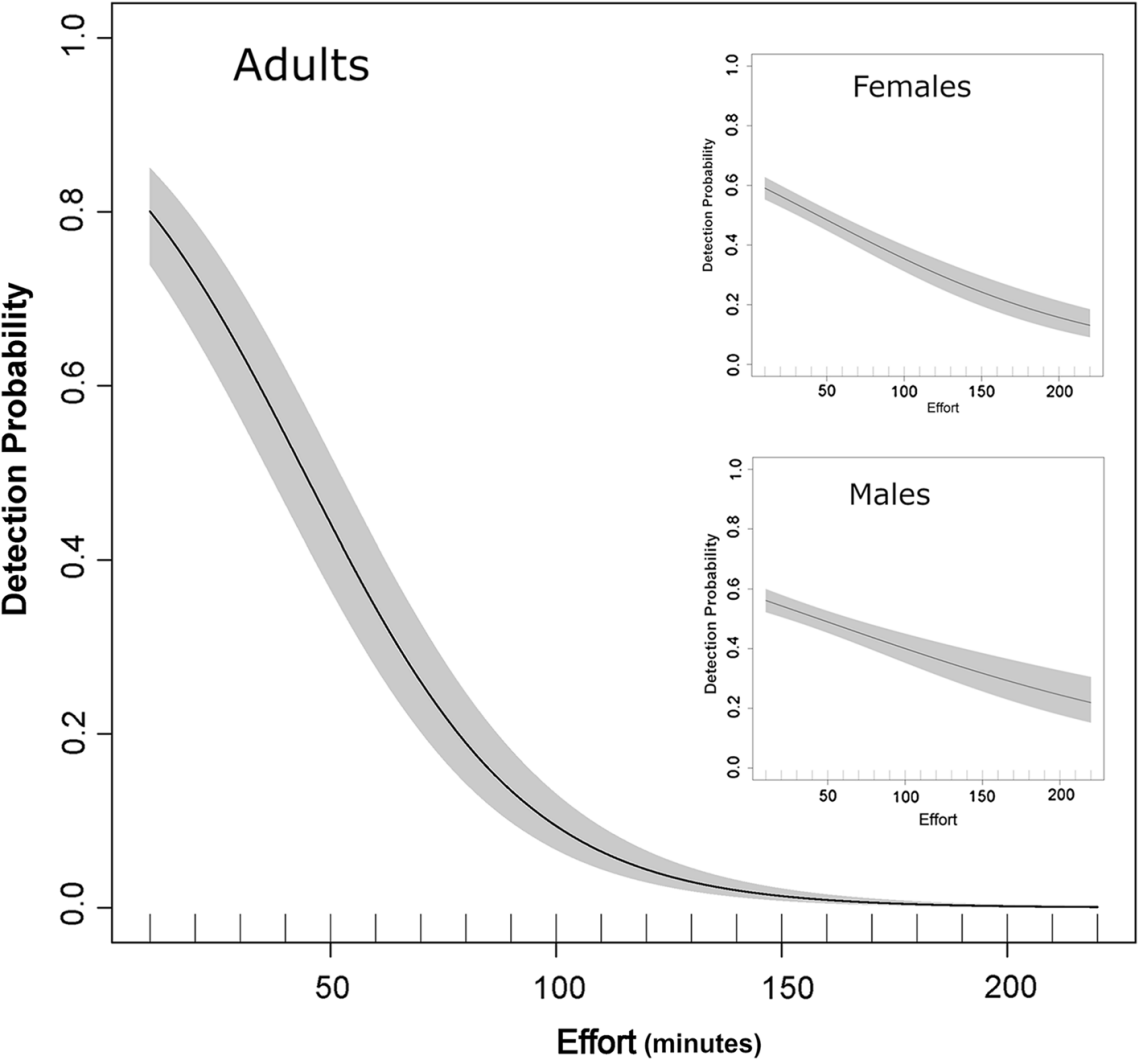
GAMM-derived non-linear association between the collection effort (measured in 10-min increments) and the probability of detecting a house infested with *Ae. aegypti* adults (main panel), males and females (inset). Non-linear terms were all statistically significant (*P* < 0.001)

**Fig. 2:**
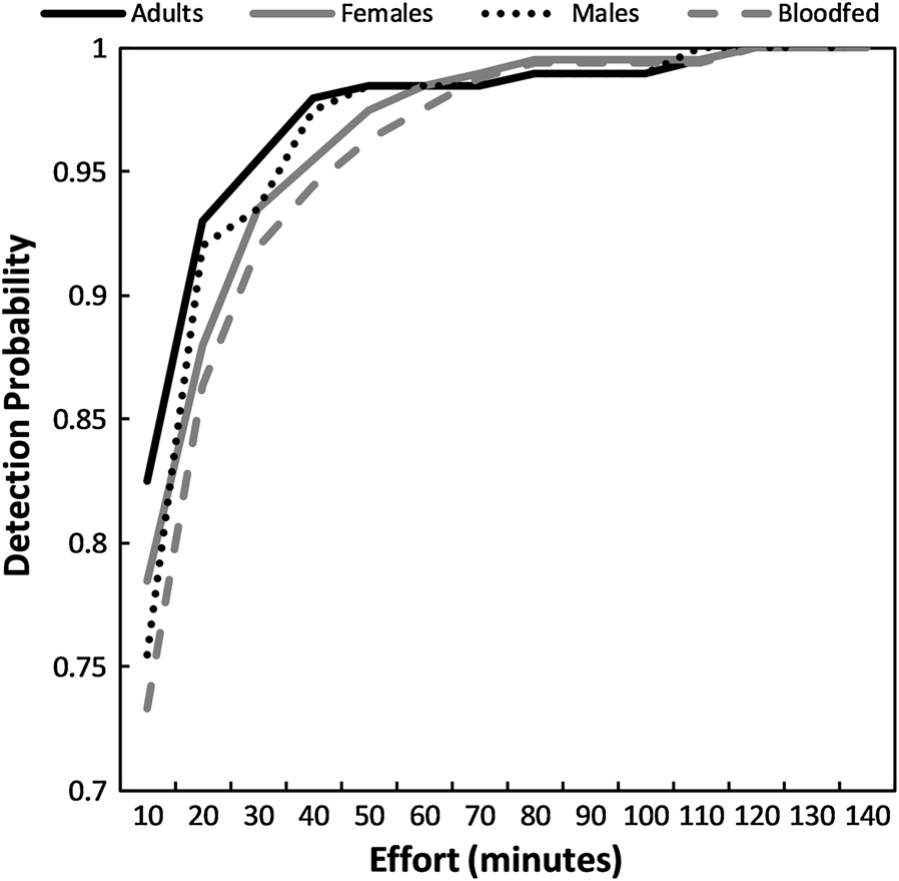
Cumulative probability of detecting an *Ae. aegypti* infested house as the collection effort is increased in 10-min increments. Probabilities were calculated for *Ae. aegypti* adults, females, males and blood-fed females

A total of 3439 *Ae. aegypti* (female to male ratio, 1.7:1) were collected in 200 houses employing a total catch effort of 269 h (a total of 1615 10-min collection rounds). All mosquitoes were collected within 16 10-min sampling rounds (up to 160 min). Most houses were infested with *Ae. aegypti* adults in at least one 10-min sampling round (*n* = 179, 89.5%), whereas 84.5% were infested with *Ae. aegypti* females, 71.5% with *Ae. aegypti* males and 79.5% with blood-fed *Ae. aegypti* females in at least one round. When analyzed by house, the probability of detecting a positive house (derived from a binomial GAMM) decreased as the population was sampled in sequential rounds for all metrics (Fig. 1), confirming the utility of sequential sampling in detecting finite populations inside houses. Sensitivity of detection of positive houses in the first sampling round was 82.5% for adults, 78.5% for females, 75.5% for males and 73.3% for blood-fed females (Fig. 2). Cumulative sensitivity increased slightly with increases in capture effort, reaching an asymptote at 40 min for any adults, 50 min for males and 60 min for females (Fig. 2). Aggregating data from the first two sampling rounds (i.e. equivalent to performing a 20-min collection) was associated with a modest increase in sensitivity (+10.5% for any adults, +9.5% for females, +16.5% for males and 13% for blood-fed females) (Fig. 2).

**Fig. 3:**
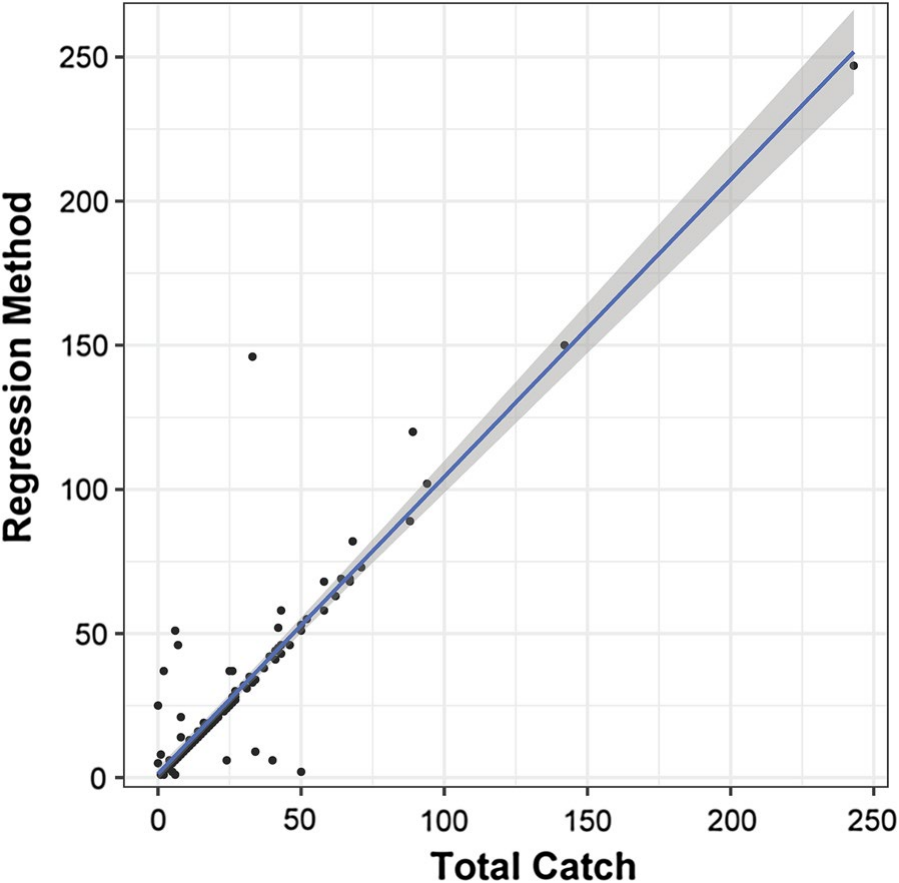
Association between total catch (sum of all *Ae. aegypti* collected across all sampling rounds) and total *Ae. aegypti* estimated by the regression method proposed by Carle and Strub [32]. Line represents maximum-likelihood fit from a simple linear regression, and gray bands indicate 95% confidence interval

**Fig. 4:**
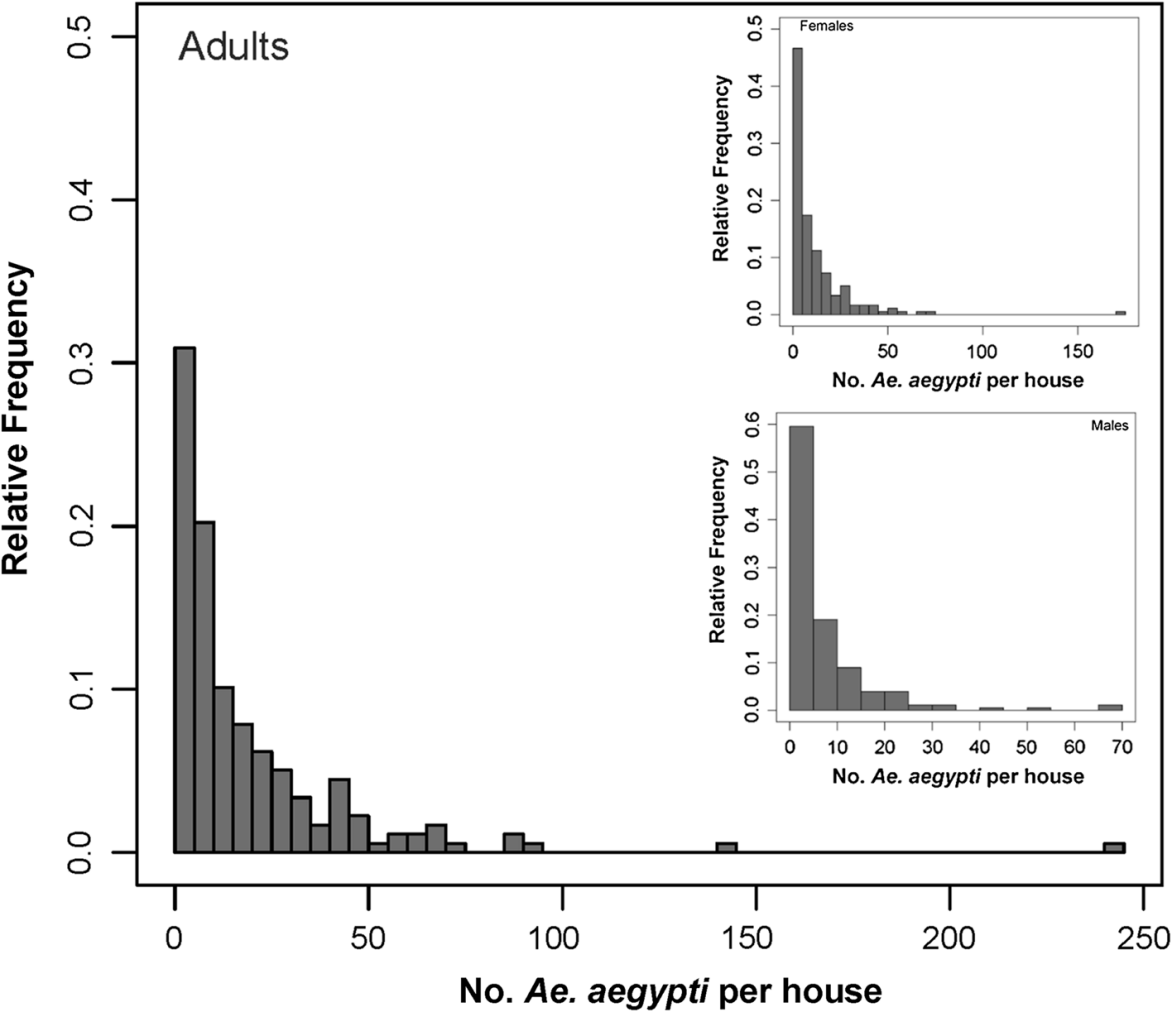
Relative distribution of the total catch of *Ae. aegypti* adults (main panel), males and females (inset) in 200 houses of Merida, Mexico

**Fig. 5:**
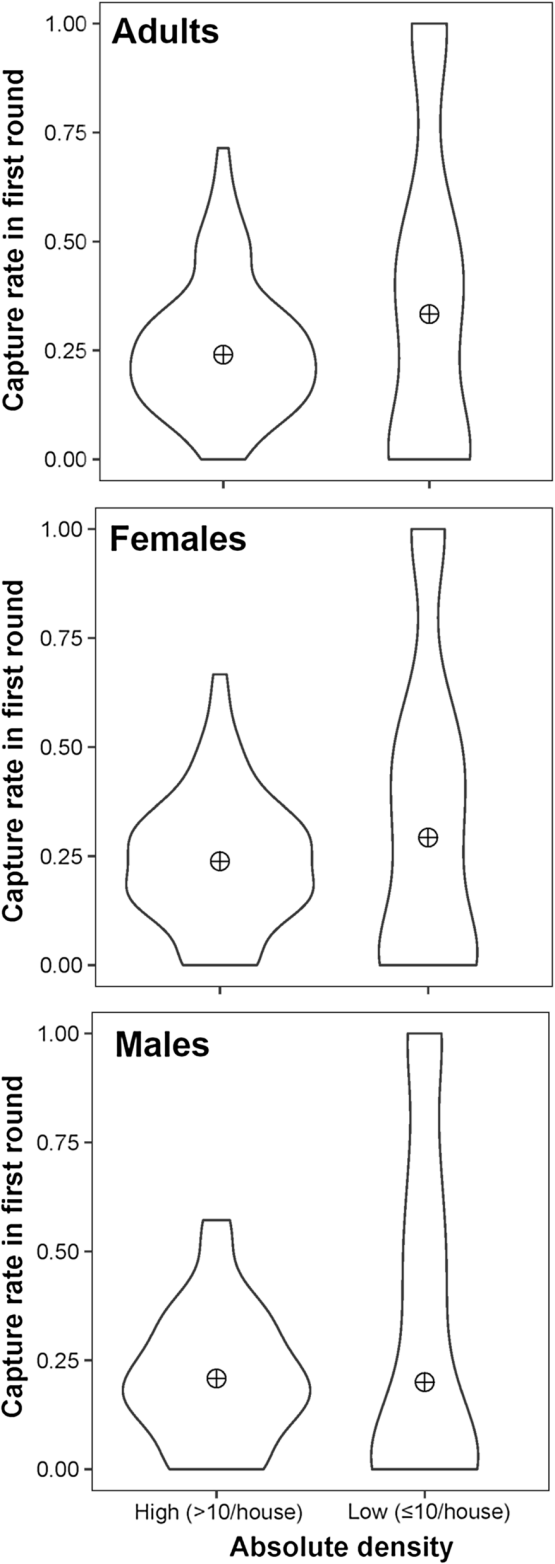
Violin plots of the median catchability (the percentage of total *Ae. aegypti* from a house that were collected on the first 10-min round) across two levels of vector absolute density (low, less than or equal to 10/house; high, greater than 10/house)

The association between the total catch (sum of *Ae. aegypti* collected across all sampling rounds) and the number of *Ae. aegypti* estimated by the regression method is shown in Fig. 3. Across all levels of *Ae. aegypti* density, both methods provided nearly equivalent results (Fig. 3). A linear regression showed that absolute adult *Ae. aegypti* density estimates from total catch and the regression method are significantly and positively associated (*beta* = 0.826; *standard error of beta* = 0.03; *t*-value = 31.6, *P* < 0.001, *R*^2^ = 0.85). As such, and for simplicity, the remainder of the manuscript will utilize total catch estimates for calibrating relative and absolute densities. Total catch was skewed (Fig. 4), averaging a total number per positive house of 19.3 (range, 1–244) *Ae. aegypti* adults, 12.9 (1–169) females, 14.3 (1–175) males and 11 (1–159) blood-fed females. These total catch averages were ~5 times higher than what was captured during the first sampling round, which was 4.4 (1–112) adults, 3.2 (1–86) females, 1.8 (1–49) males and 2.3 (1–65) blood-fed females. The absolute density of *Ae. aegypti* females and blood-fed females per house were strongly and significantly associated (linear regression, F_(1,176)_ = 10188, *P* < 0.0001, *R*^2^ = 0.98). Of the total *Ae. aegypti* collected per house, a median of 26% were captured during the first 10-min round in low density houses and 24% in high density houses (Fig. 5). While the distribution of catchability values was wider for low than for high density houses (Fig. 5), the median did not vary statistically between the two density strata for *Ae. aegypti* adults (two-sample Wilcoxon, W = 3568, *P* = 0.2379), females (W = 2932, *P* = 0.2532) or males (W = 1870, *P* = 0.7341) (Fig. 4). Differences among houses in percent catchability were not explained by neither the percentage of doors nor windows with mosquito screens (Gaussian generalized linear model, *beta* = −0.15, *z* = −0.368, *P* = 0.71), the area of the house (*beta* = 0.003, *z* = −0.73, *P* = 0.47) the number of residents in the house (*beta* = −0.07, *z* = −0.90, *P* = 0.38) and whether the house had low (≤10/house) or high (>10/house) absolute *Ae. aegypti* density (*beta* = −0.49, *z* = −1.42, *P* = 0.16). Given the lack of significant association between abundance and capture rate, further analyses considered abundance as a continuous variable.

**Fig. 6:**
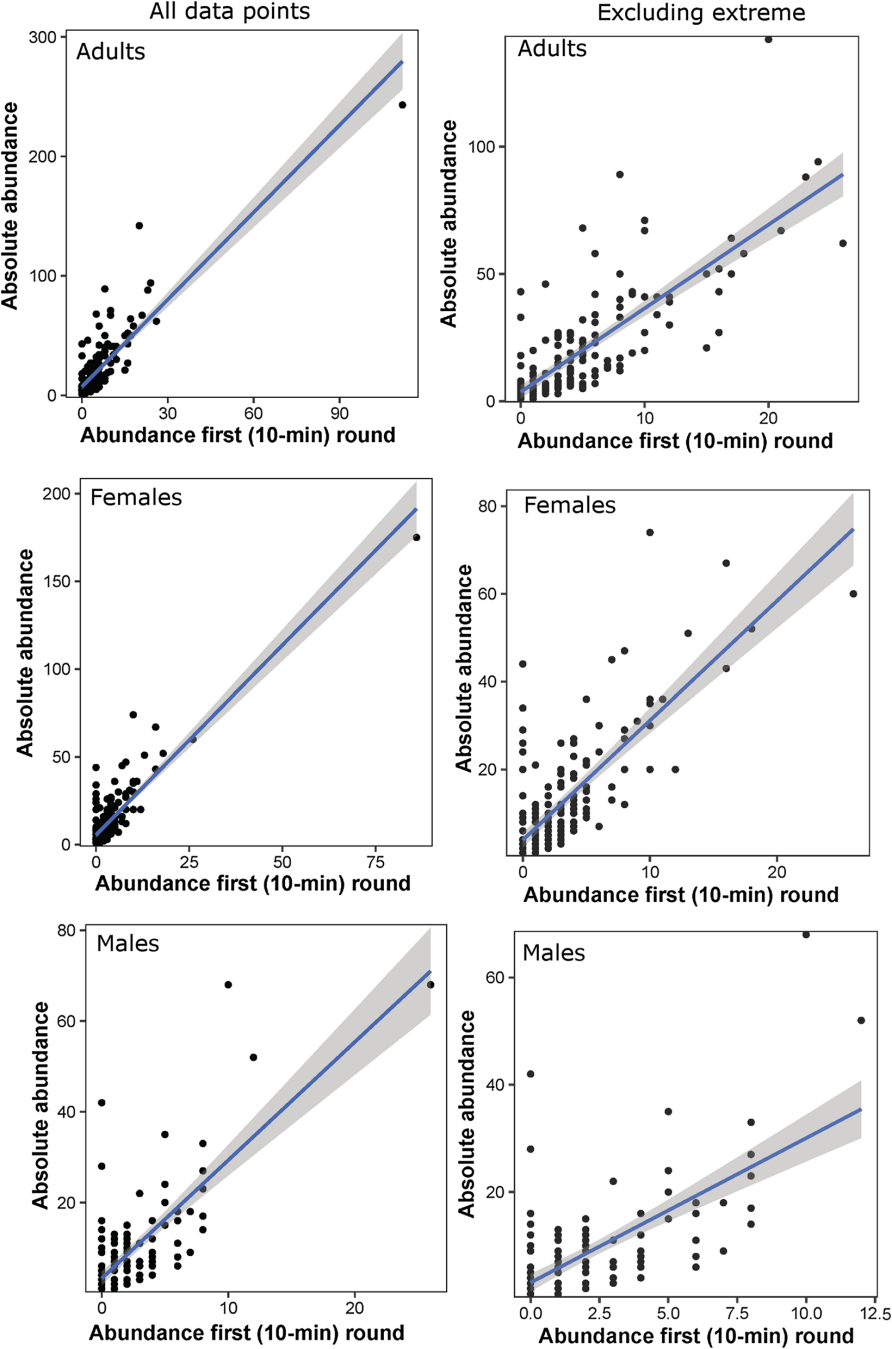
Linear relationship between the relative abundance (number collected on first 10-min round) and total catch (total collected across all sampling rounds) of total *Ae. aegypti* adults, females and males indoors. Line and 95% CI are based on regression equation parameters provided in Table 1

There was a significant positive linear relationship between the relative abundance of *Ae. aegypti* collected in the first 10-min round and the total catch for all adult metrics (Table 1, Fig. 6). The strong association between relative and absolute abundance allowed for the estimation of calibration coefficients from simple linear regressions (Table 1). Given the presence of a high capture location where 243 adults were collected, we implemented regression models including and excluding this high infestation location. Excluding the highest collection point from the regressions (Fig. 6, right panel) reduced model fit from an *R*^2^ of 0.73 to an *R*^2^ of 0.62 for adults and 0.76 to 0.59 for females while *R*^2^ for males remained unchanged. The intercept in the models including all data points ranged from 7.1 ± 1.2 for adults, 5.5 ± 0.8 for females and 3.3 ± 0.8 for males (Table 1). These metrics are indicative of the sampling error associated with a single 10-min round of adult collection and allow for adjusting, through the regression equation, the capture rate of indoor *Ae. aegypti* when at least one mosquito is collected within the initial 10-min collection.

**Table 1:** Correction coefficients to calculate absolute *Ae. aegypti* density for each of the three metrics (adult, female and male) derived from fitting a simple linear regression to total catch data (*y*) and the number collected during the first 10-min sampling round (*x*), and following the equation y = *a* + b*x*, where a is the intercept. The top panel shows the fit to all data and the bottom panel shows the fit to the dataset excluding the extreme values shown in Fig. 4

Density metric	Parameter	Estimate	SE	*t*-value	*P*-value
All data points					
Adults	Intercept	7.1994	1.1895	6.052	<0.0001
	Abundance first round	2.4324	0.1106	21.983	<0.0001
Females	Intercept	5.46176	0.77034	7.09	<0.0001
	Abundance first round	2.16220	0.09413	22.97	<0.0001
Males	Intercept	3.2710	0.7628	4.288	<0.0001
	Abundance first round	2.6053	0.2008	12.973	<0.0001
Excluding extreme point					
Adults	Intercept	3.6833	1.2974	2.839	0.00506
	Abundance first round	3.2827	0.1926	17.041	<0.0001
Females	Intercept	3.9061	0.8541	4.573	<0.0001
	Abundance first round	2.7259	0.1777	15.336	<0.0001
Males	Intercept	3.1144	0.8208	3.795	<0.001
	Abundance first round	2.6939	0.2626	10.258	<0.0001

*Abbreviation*: SE, standard error

## Discussion

While entomological sampling is a cornerstone of vector-borne disease research and surveillance, there is a paucity of information about the sensitivity of existing sampling methods. Our research provides detailed estimates of the sensitivity of using Prokopack adult mosquito aspirators to collect indoor *Ae. aegypti*. Our estimates show that, regardless of the absolute abundance and housing characteristics, a 10-minute collection detects ~80% of houses that are infested with *Ae. aegypti* and captures 24–26% of all *Ae. aegypti*. Our data was applied to a simple mathematical equation to provide the basis for future calibrations of relative density obtained from 10-minute collections into absolute abundance estimates of indoor *Ae. aegypti*.

While relative measures of vector density can be informative and allow for comparisons between different time points and locations, they can also suffer from collector or sampling errors and are prone to spatial and temporal bias [1–3]. Identifying a functional relationship between estimates of relative and absolute density has been common practice in agriculture [4] and wildlife research [5]. To date, the advantage and power of quantifying absolute population abundance has not been widely employed in the field of medical entomology. For *Ae. aegypti*, the only example of calibration is the one performed by Johnson et al. [17] using a mini mark-release study to calibrate BG sentinel traps. Our study introduces a simpler methodology, sequential removal sampling, to reliably estimate calibration coefficients to quantify absolute *Ae. aegypti* density indoors. This methodology has the potential to be expanded to other disease vectors readily collected using Prokopack aspirators such as endophilic *Anopheles* spp. [19–21] and *Culex* spp. [22]. While our study shows the benefit of sequential removal sampling to quantify indoor absolute density, the applicability of this methodology for outdoor *Ae. aegypti* collections will need to be thoroughly assessed. A study in Cairns, Australia, found that BG-Sentinel traps collected more *Ae. aegypti* than CDC Backpack aspirators outdoors [35]. As such, Prokopack removal sampling outdoors will have to be implemented at a much higher effort than indoors, and probably with lower collection efficiency compared with traps. Given the expected low yield per sample, implementation of the regression method for estimating absolute density would be a necessary step, provided sequential collections lead to a reduction in the catch per unit effort over time [1].

The finding of a decay in catchability with each sampling round (Fig. 1) supports a key removal sampling assumption: populations are closed at the time of sampling. Using simple linear regressions, we estimated correction coefficients, which allowed calculation of absolute *Ae. aegypti* numbers indoors at the time of collection (together with 95% confidence intervals). We generated two fits to the data, one including and another excluding a house where more than 240 *Ae. aegypti* were collected. The model fit the data better when using all data points, but we suggest that our equations could be applied as follows: for houses where fewer than 30 total *Ae. aegypti* are collected during the ten-minute collection round, the equations excluding the extreme data point should be used. For houses where more than a total of 30 *Ae. aegypti* are collected, the equation including all data points should be used. In either occasion, houses with no *Ae. aegypti* should be excluded from the calculation, as including them would lead to estimates of density equal to the intercept of the model. We believe that applying the regression equation estimated from our study would allow a more precise estimation of absolute abundance from a single 10-minute collection round. As similar sequential removal studies are performed in other locations (ideally with different housing characteristics and vector densities), the information generated will help refine model equations and increase the value of calibrated adult *Ae. aegypti* indices.

**Fig. 7:**
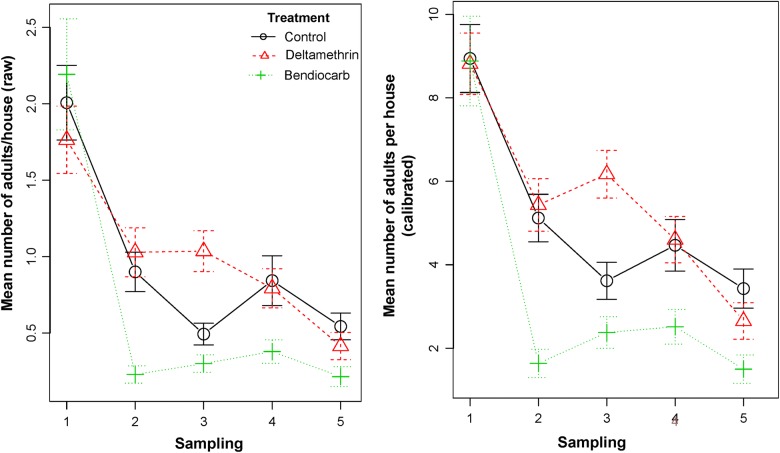
Data from a randomized controlled trial evaluating the impact of indoor residual spraying on *Ae. aegypti* [36]. The left panel shows the (raw) relative density data (from 10-min collection) and the right panel shows the same data after calibration to estimate the absolute density of *Ae. aegypti* using the linear regression equation and parameter values from Table 1 (top panel, using all data points)

Using absolute (rather than relative) density estimates has the potential to change the way entomological data are interpreted when evaluating public health interventions. As an example, we used the calibration coefficient generated by this study to calculate total indoor adult *Ae. aegypti* absolute abundance at baseline and at four sampling time points after applying indoor residual spraying (Fig. 7, data from [36]). Using the estimates of absolute abundance provided a much more dramatic measure of the absolute impact of indoor residual spraying, with an estimated reduction from ~10.0 to ~2.0 *Ae. aegypti* per house, compared to 2.0 to 0.25 *Ae. aegypti* per house when using the relative abundances before calibration (Fig. 7). While the relative change is still proportional among both sets of data, the absolute impact may be more relevant at the time of identifying entomological thresholds of protection or intervention impact. Rear-and-release methods of vector population suppression or modification (e.g. sterile insect technique, *Wolbachia*-related interventions, transgenic mosquitoes) would also benefit from more accurate estimates of absolute mosquito abundance. As mosquito releases are informed by the relative density of adults sampled (e.g. 10 times more adults than the ones collected in weekly BG-sentinel trapping [37]), estimates of absolute population density are key for successful rear-and-release implementation. Our estimates indicate that houses can have up to five times more adult *Ae. aegypti* than estimated with a 10-minute collection round. As such, estimates of released mosquitoes based on relative density would significantly underestimate the effort needed for successful population control or modification.

Compared to other species, *Ae. aegypti* is considered a low abundance vector [8]. This assessment has been inferred from the low numbers of adult *Ae. aegypti* generally collected indoors using aspirators, with collections during high density periods averaging four to five females per house [11, 16]. Our estimates of absolute indoor *Ae. aegypti* density show a different picture. On average, ~13 female *Ae. aegypti* were collected per house, with a highly overdispersed distribution that, in our sample of 200 houses, reached a maximum of 169 females. In fact, 21.4% of *Ae. aegypti* positive houses had 20 or more females indoors. In Merida, the average household size is four people. Assuming equal biting probability, we can hypothesize that in 21% of houses with *Ae. aegypti*, there are a minimum of five *Ae. aegypti* females per person. Mathematical models have been a useful tool for linking such entomological measures to virus transmission estimates. However, most models have relied on relative density data to parameterize mosquito abundance [38]. As more reliable estimates of *Ae. aegypti* absolute density are generated, there will be a need to recalculate arbovirus transmission risk under scenarios that, based on our estimates, could include up to five times more *Ae. aegypti* females than previously assumed. Whether these updated estimates of vector abundance will result in profound epidemiological changes will have to be further explored.

For mosquito-borne pathogens, there is a renewed interest in comprehensively evaluating the epidemiological impact of vector control [39, 40]. An emphasis on epidemiological end-points for evaluating interventions has emerged from decades of research pointing to a poor association between entomological measures and vector-borne disease transmission risk [39, 41]. Particularly for *Aedes*-borne viruses, poor estimates of *Ae. aegypti* adult abundance, combined with the focal and local nature of virus transmission [42, 43] have challenged the finding of an association between entomologic data and epidemiological risk [8]. Our findings suggest that these poor correlations may also be the result of a lack of accuracy in estimating the absolute abundance of *Ae. aegypti*. As such, we recommend a greater emphasis on applying methods to estimate total adult *Ae. aegypti* density in future entomological studies and vector control evaluations.

## Conclusions

While myriad sampling methods are used to quantify the density of vectors (e.g. traps, active collections, landing counts), estimates of their sensitivity are rare. This knowledge gap impacts the reliability of entomological estimates, and limits the quantification of the entomological impact of vector control. In this article, we introduce a methodology for rapidly estimating the absolute density of vectors indoors, and use it to estimate the sensitivity of the Prokopack mosquito collector on *Aedes aegypti* populations from Merida, Mexico.

## Data Availability

Data supporting the conclusions of this article are included within the article. The datasets used and/or analyzed during the present study are available from the corresponding author upon reasonable request.

## Abbreviations

CPUE: catch per unit effort
MOH: Ministry of Health
